# Peer Review of “Satisfaction With Health Care Services at the Pediatric Specialist Clinic of the National Referral Center in Malaysia: Cross-sectional Study of Caregivers’ Perspectives”

**DOI:** 10.2196/37051

**Published:** 2022-05-25

**Authors:** Bruno José Nievas-Soriano

**Affiliations:** 1 Nursing, Physiotherapy, and Medicine Department University of Almeria Almeria Spain

**Keywords:** pediatrics, caregivers, health care services, public hospital, Malaysia, public-private-partnership, children

*This is a peer-review report submitted for the paper "Satisfaction With Health Care Services at the Pediatric Specialist Clinic of the National Referral Center in Malaysia: Cross-sectional Study of Caregivers’ Perspectives."*

## Round 1 Review

### General Comments

This paper [1] describes interesting research about factors affecting the satisfaction of caregivers at a national referral center. I really liked the research performed and the article. Nevertheless, I think that there are some minor aspects that perhaps could be better described so the readers can better understand the results and their external validity. The authors do explain the limitations adequately, but perhaps some aspects could be clarified within the main text of the article.

### Specific Comments

#### Major Comments

1. In Methods, the authors write that “This cross-sectional study was conducted at the Tunku Azizah Hospital, Kuala Lumpur, Malaysia. Subjects were caregivers to children seen with an appointment at the clinic.” They also write that “This study was conducted at the hospital’s Paediatric Specialist Clinic by convenience sampling. Self-administered, structured questionnaires were distributed to consenting participants. Subjects who agreed to participate were given questionnaires after seeing the doctor and while waiting for the date of their next consultation.”

Selection bias is probably the most important limitation of this research. Selection bias is almost unavoidable, so the authors must make a considerable effort to clearly describe where they obtain the sample from, so the readers can have a clear idea of the main features of that sample, which also should be described. To better understand the results (and therefore the conclusions), it would be very interesting to know, in more detail, how the patients were chosen, the attrition rate, or other factors related to the sample selection. Therefore, I would propose that the authors better describe where the sample is obtained from and how they were chosen.

2. In that same section, the authors write that “A total of 600 questionnaires distributed to the clinic, and we received 502 responses, giving a rate of 83.7%. Of these 502 responses, 43 were unusable and were excluded from this study, and the remaining 459 (91.4%) questionnaires were analysed. Some 2,238 patients were registered for an appointment at the clinic during this data collection period.”

It would be interesting if they describe in the article if they performed any sample size estimation and which method did they employ, in that case.

3. The authors write that “This was part of a hospital-level survey assessing satisfaction among caregivers attending the clinic using the SERVQUAL instrument.”

They properly describe the dimensions of the questionnaire, but perhaps it would be useful to know if this tool has been validated (or has required transcultural adaptation) to be used with this specific sample.

Despite these aspects, which are easily solvable, I think that this is a very interesting article that can be useful for other researchers.

#### Minor Comments

Some sentences and some paragraphs are perhaps a bit too long, and therefore, they are a bit confusing to read, but overall, the article is very well written.

## References

[1] Selvarajah T, Yamamoto E, Saw YM, Kariya T, Hamajima N (2022). Satisfaction with health care services at the Pediatric Specialist Clinic of the National Referral Center in Malaysia: Cross-sectional study of caregivers' perspectives. JMIRx Med.

